# Cross-cultural adaptation and psychometric validation of the Chinese quality-of-life questionnaire for patients with systemic sclerosis

**DOI:** 10.7717/peerj.20331

**Published:** 2025-11-19

**Authors:** Yinfeng Hu, Wenjie Zhong, Liqiong Zhou

**Affiliations:** 1 Department of Rheumatology and Immunology, South China Hospital, Medical School, Shenzhen University, Shenzhen, Guangdong, China; 2 Department of Rheumatology and Immunology, The First Affiliated Hospital of Henan University of Science and Technology, Luoyang, Henan, China

**Keywords:** Systemic sclerosis, Quality of life, Questionnaire, Scale, Psychometric evaluation, Cross-cultural adaptation

## Abstract

**Background:**

Patients with systemic sclerosis (SSc) frequently experience symptoms such as pain, fatigue, and functional impairments, leading to a significant decline in their quality of life (QoL). Accurately evaluating the QoL of SSc patients is crucial for developing personalized treatment plans and enhancing prognosis. The Systemic Sclerosis Quality of Life Questionnaire (SScQoL), an internationally recognized SSc-specific tool, is commonly used for QoL assessment. However, this questionnaire has not undergone cross-cultural adaptation in China, and its reliability and validity require further validation.

**Objectives:**

To localize the Systemic Sclerosis Quality of Life Questionnaire (SScQoL) into Chinese and assess its reliability and validity.

**Methods:**

Following the principles of scale introduction, the Beaton model was employed to translate and back-translate the English version. The scale underwent cross-cultural adaptation through expert consultation, resulting in the Chinese version of the SScQoL test. From August 2023 to December 2024, a convenience sampling method was used to recruit 160 patients with systemic sclerosis from two hospitals for questionnaire surveys to evaluate questionnaire reliability and validity.

**Results:**

SScQoL comprises five dimensions and 29 items. The content validity index (CVI) at the item level ranges from 0.83 to 1.00, and the CVI at the scale level is 0.97. Exploratory factor analysis identified five common factors, with a cumulative variance contribution rate of 65.761%. The Cronbach’s α coefficient for the total scale is 0.922, and the Cronbach’s α coefficients for the five dimensions range from 0.756 to 0.942. The test-retest reliability for the total scale is 0.969; for the five dimensions, it ranges from 0.710 to 0.961.

**Conclusions:**

SScQoL is a reliable and effective tool for evaluating patients’ quality of life with systemic sclerosis. This tool can support nurses and researchers and help them formulate targeted strategies, thereby significantly improving patients’ quality of life with systemic sclerosis.

## Introduction

Systemic Sclerosis (SSc), also known as scleroderma, is a rare, highly pathogenic autoimmune disease marked by extensive fibrosis and vascular abnormalities in the skin, joints, and internal organs, especially the esophagus, lower gastrointestinal tract, lungs, heart, and kidneys (Barsotti, Stagnaro & Della Rossa, 2015). As the disease progresses, patients may experience complications, such as joint stiffness, impaired pulmonary function, cardiac issues, and gastrointestinal problems. These complications affect physical health, mental well-being, and social functioning, significantly lowering overall quality of life (Sakkas et al., 2018; Chrabaszcz et al., 2020). Therefore, assessing quality of life is essential in managing SSc.

Quality of life is a comprehensive indicator that reflects an individual’s health status. It refers to how individuals perceive their social position and living conditions based on personal standards, values, and goals within a given cultural context (Shenbin, 2017). A 2021 US online survey found (Chen et al., 2023) that more than one-third of systemic sclerosis patients were dissatisfied with their quality of life, and over a quarter felt pessimistic about the future. Clinical guidelines for diagnosing and treating systemic sclerosis state (Adigun, Goyal & Hariz, 2024) that the treatment goal is to improve patients’ quality of life and prevent further organ damage.

Evaluating quality of life in systemic sclerosis patients reveals the disease’s multidimensional impact on physical function, mental health, and social adaptation. It offers a scientific basis for creating personalized treatment plans to improve patient health. When clinical symptom improvement is limited, tracking quality of life has become a key part of clinical practice and an essential indicator of treatment effectiveness (Adigun, Goyal & Hariz, 2024). Quality-of-life assessments also help identify psychological issues like depression and anxiety early, enabling timely mental health support. Research further shows that including quality of life data in broader evaluations helps quantify the disease’s burden on families and society, supports resource allocation decisions, and improves patient satisfaction and treatment adherence (Xianhao, 2021).

Internationally, several general scales have been developed to evaluate quality of life in patients with systemic sclerosis, such as the Euro Qol five dimensions questionnaire (EQ-5D), the 36-Item Short Form Health Survey (SF-36), and the Scleroderma Health Assessment Questionnaire (SHAQ) (Sarac et al., 2023). However, these scales are not specific to systemic sclerosis, which may limit their accuracy in assessing the disease’s impact on patients’ quality of life. Naomi Reay (2008) developed the Systemic Sclerosis Quality of Life Questionnaire (SSc-QoL) to address this issue in 2008. It includes five domains—pain, sleep, function, emotion, and social interaction—with 29 items. The scale was validated using the Rasch model, showing strong psychometric properties. The SSc-QoL has been translated into multiple languages, including Arabic, French, German, Italian, Polish, Spanish, Swedish, and Turkish, and has shown good reliability and validity in all versions (Sarac et al., 2023). However, no validated Chinese version currently exists. This study aims to develop a reliable and culturally adapted Chinese version of the SSc-QoL through translation, back-translation, and cultural adaptation, providing Chinese healthcare professionals with a standardized assessment tool.

## Materials and Methods

**Figure 1:**
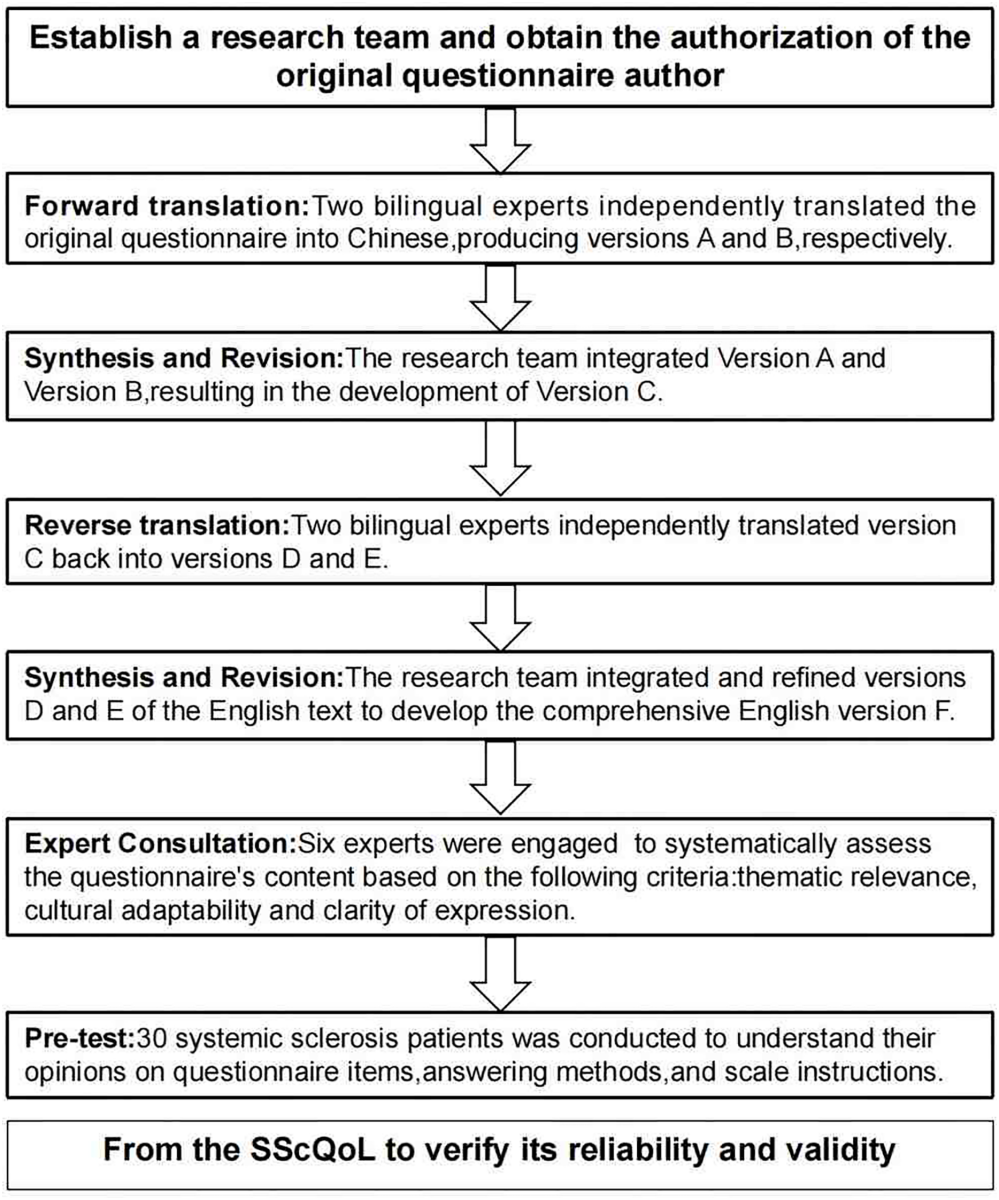
Cross-cultural steps of SScQoL. Note: SScQoL, Systemic Sclerosis Quality of Life Questionnaire.

The author has secured permission from the original author to utilize the questionnaire. This study adheres to the Beaton model (Beaton et al., 2000, 2007), which comprises six stages: forward translation, translation synthesis, back-translation, back-translation synthesis, expert correspondence, and pilot study (see Fig. 1: Intercultural Steps of SSC-QOL). The reporting of this study conforms to the International Society for Pharmacoeconomics and Outcomes Research (ISPOR) criteria proposed by Wild et al. (2005).

### Content validity

A total of six nursing research experts will be selected. Of these six experts, the highest educational qualification is a doctorate, while the lowest is a bachelor’s degree. The selection criteria for these experts are as follows: (1) they must possess at least a bachelor’s degree and hold a deputy senior title or higher as physicians or nurses; (2) they should have over 10 years of experience working in the field of rheumatology and immunology diseases; (3) they agree to participate in this study. Invite experts to suggest modifications in three areas: cultural adaptability, clarity of language expression, and relevance of questionnaire content. The questionnaire content is assessed using a 4-point Likert scale. The scoring criteria are defined as follows: one point indicates “very irrelevant,” two points indicate “somewhat irrelevant,” three points indicate “relevant,” and four points indicate “very relevant.” Detailed information on the experts’ backgrounds and specific evaluation results can be found in Table 1.

**Table 1:** General information on the experts and the detailed evaluation outcomes.

No.	Gender	Age/Working year	Major	Highest academic degree	Familiarity	Basis for judgment and degree of influence			
						Theoretical analysis	practical experience	Reference	Subjective judgment
1	Female	58/36	Nursing	Bachelor’s	Very familiar	Large	Large	Large	Small
2	Female	49/25	Nursing	Master	Familiar	Large	Medium	Large	Small
3	Male	46/21	Clinical	Doctor	Very familiar	Large	Large	Medium	Small
4	Female	43/15	Nursing	Master	Very familiar	Large	Large	Large	Medium
5	Male	55/29	Clinical	Doctor	Very familiar	Large	Large	Large	Small
6	Male	53/30	Clinical	Doctor	Very familiar	Large	Large	Large	Medium

### Translation process

#### Forward translation process

The researchers sought permission from the original authors *via* email to translate the English version into Chinese. Following Beaton’s translation method (Beaton et al., 2000, 2007), two bilingual individuals proficient in Chinese and English independently conducted forward translations, including a nursing graduate student with experience studying in the UK and a nursing master’s student. Drafts A and B were generated, and after careful comparison and analysis, the research team produced an initial high-quality translated version, draft C.

#### Back translation of the initial translated version C

An English master’s student and a nursing doctoral student independently conducted the back translation of the initial version C into English, ensuring the quality of the process. Both individuals were unaware of the original scale and its specific translation procedures, resulting in translations D and E. The research team and bilingual experts thoroughly discussed translations D and E, ultimately reaching a consensus to create an integrated back translation version F. Version F was then sent *via* email to the original author for evaluation regarding any potential alterations in meaning from the original scale, as well as to request modification suggestions.

### Pilot study

A preliminary survey was conducted on 30 patients with systemic sclerosis. Participants were asked about their understanding of the questionnaire items, how to answer them, and the rating scale instructions.

### Field test of the questionnaire

Convenience sampling was employed to recruit 160 patients diagnosed with systemic sclerosis admitted for treatment in the rheumatology and immunology departments of two hospitals in Shenzhen and Luoyang between August 2023 and December 2024. The study objectives and methods were thoroughly explained to the participants, and their informed consent was obtained before administering the questionnaire. Inclusion criteria encompassed: (1) fulfillment of the diagnostic and classification criteria for systemic sclerosis jointly established by the American College of Rheumatology (ACR) and European League Against Rheumatism (EULAR) in 2013; (2) consciousness and practical communication ability; (3) signing an informed consent form. Exclusion criteria comprised: (1) the presence of mental disorders; (2) the occurrence of severe complications such as cardiovascular, cerebrovascular, or renal diseases. Considering statistical analysis requirements, a minimum sample size should be five to ten times greater than the number of items (Sousa & Rojjanasrirat, 2011). As there were a total of 29 items on the questionnaire used in this study, potentially leading to a rate of invalid questionnaires up to 20%, we calculated that a minimum sample size required would be *N* = (29 × 5) × (1 + 20%) = 174; while the maximum sample size needed would be N = (29 × 10) × (1 + 20%) = 252. Due to systemic sclerosis being classified as a rare disease entity, ultimately, we distributed 160 questionnaires.

The researchers used the General Information Questionnaire and the SSc-QoL Questionnaire in this study. Investigators received systematic training before data collection to ensure the survey was conducted smoothly. The training covered the research objectives, methods, questionnaire procedures, and key precautions. Paper-based questionnaires were distributed in person, completed on-site, and returned immediately. Researchers ensured no confusing or misleading language was used during completion to maintain data quality and provide authentic, objective responses.

### Statistical analysis

Microsoft Excel 2010 was used for data collation. Two individuals cross-validated data entry using SPSS 22.0 (Armonk, NY, USA) for comprehensive data analysis. Quantitative data were presented as frequency and percentage (%). Item analysis was conducted to assess the suitability and reliability of questionnaire items, providing a basis for item selection or modification. The critical ratio (CR) and correlation coefficient were used for item analysis (Rongrong & Yiyu, 2024).

Critical Ratio (CR): the systemic sclerosis questionnaire scores were sorted in ascending order. The lowest 27% were classified as the low-score group and the highest 27% as the high-score group. Independent t-tests were conducted on each item between the two groups. Items with no significant difference (*P* > 0.05) or a CR < 3.0 may be excluded (Li & Liu, 2018). Correlation coefficient: each item’s score correlated with the total scale and dimension scores. Items with r ≥ 0.3 (*P* < 0.05) were retained (Weiling et al., 2023). Reliability was assessed using three indicators: i. Cronbach’s α coefficient: a value above 0.8 indicates good internal consistency. ii. Split-half reliability: a coefficient above 0.8 indicates strong internal consistency. iii. Test-retest reliability: twenty systemic sclerosis patients completed the questionnaire twice, 1 week apart. A Pearson correlation coefficient above 0.7 indicates good test-retest reliability (Wu, 2010). The significance level was set at α = 0.05. Validity was evaluated through content and construct validity. Content validity: six experts rated each item’s relevance to quality of life on a 4-point Likert scale (1 = not relevant, 4 = strongly relevant). The item-level content validity index (I-CVI) is ≥0.78, and the average scale-level content validity index (S-CVI/Ave) is ≥0.90, which indicates satisfactory content validity (Shuhua et al., 2024). Construct validity: exploratory factor analysis was performed using principal component analysis and orthogonal rotation. Factors with eigenvalues >1 were extracted. Items with factor loadings >0.4 and a cumulative variance >50% indicated good construct validity (Weiling et al., 2023). Before factor analysis, Kaiser-Meyer-Olkin (KMO) and Bartlett’s tests were conducted. A KMO > 0.8 and significant Bartlett’s test indicated suitability for factor analysis (Zhang et al., 2022). The ceiling effect occurs when many subjects score near the maximum, indicating low sensitivity for high-ability individuals. Floor effect occurs when many scores are near the minimum, indicating low sensitivity for low-ability individuals. Either effect is considered significant when more than 15% of subjects are affected (Terwee et al., 2007).

### Ethical considerations

This study received approval from the Ethics Committee of South China Hospital of Shenzhen University in compliance with the Declaration of Helsinki. All methods followed the relevant guidelines and regulations—approval number-(Ethical No. 20241121002-XZ01). Written informed consent was obtained from all individual participants involved in this study.

## Results

### The result of cross-cultural adaptation

The experts recommend revising item 2 from “I have had it on my mind (it refers to being sick-systemic sclerosis)” to “I have consistently kept this condition, systemic sclerosis, at the forefront of my mind.”; revising item 8, “I feel that I am fighting all the time,” to “I feel like I am constantly facing challenges every moment,” and revising item 21, “I feel very isolated,” to “The current situation makes me feel quite lonely.” Refer to Table 2 for modification of the SScQoL items.

**Table 2:** Modification of the SScQoL items, expert scoring, and calculation of content validity.

		Expert rating						Experts scoring 3 or 4 points	I-CVI	S-CVI
No.	Item	A	B	C	D	E	F			
Q1	I have to think carefully about everything I do.	4	4	4	3	4	4	6	1	0.97
Q2	I have had it on my mind (it refers to being sick - systemic sclerosis)	4	4	4	4	3	4	6	1	
	Pre-test comments: There are issues with the expression of language.									
	Modification results: I have consistently kept this condition, systemic sclerosis, at the forefront of my mind.									
Q3	The thought of letting others down is causing me concern.	4	4	3	4	4	4	6	1	
Q4	I find my current situation frustrating	2	4	3	3	4	4	5	0.83	
Q5	I feel quite uncomfortable when I cannot accomplish tasks.	4	4	4	4	4	4	6	1	
Q6	I sometimes feel frustrated.	4	4	3	4	4	4	6	1	
Q7	I cannot expect what my tomorrow will be like.	4	3	3	4	4	4	6	1	
Q8	I feel that I am fighting all the time	4	4	4	4	3	4	6	1	
	Pretest comments: There are issues with the language translation. It is difficult to understand.									
	Modification results: I feel like I am constantly facing challenges every moment.									
Q9	My situation means that my sleep has been disrupted.	4	3	3	4	4	4	6	1	
Q10	It has a significant impact on my social life.	4	4	4	3	4	4	6	1	
Q11	The health of people around me has been impacted.	4	3	3	4	4	3	6	1	
Q12	My hands are not as dexterous as they used to be.	3	4	4	4	4	4	6	1	
Q13	It has limited my interpersonal relationships.	3	4	4	3	3	4	6	1	
Q14	I should make it a point to take more frequent breaks and rest.	4	4	4	4	2	4	5	0.83	
Q15	Every activity poses its challenges.	4	3	4	4	4	2	5	0.83	
Q16	I avoid attending certain social occasions because I feel awkward.	4	4	4	4	4	4	6	1	
Q17	The things that never bothered me have started to make me worry.	4	3	3	4	4	4	6	1	
Q18	How life is now is different from what it used to be.	4	4	4	4	4	4	6	1	
Q19	The situation is overwhelming for me.	4	3	4	4	3	4	6	1	
Q20	Having a poor sleep has a significant impact on me.	4	3	4	4	4	4	6	1	
Q21	I feel very isolated.	4	4	4	3	4	4	6	1	
	Pretest comments: There are issues with the expression of language.									
	Modification result: The current situation makes me feel quite lonely.									
Q22	Doing household chores can sometimes be challenging.	2	3	3	4	4	4	5	0.83	
Q23	I have to give up some of my hobbies.	4	4	4	4	4	3	6	1	
Q24	I feel remorseful for falling ill.	4	4	4	4	4	4	6	1	
Q25	I managed to finish washing up with incredible difficulty.	3	4	4	4	4	3	6	1	
Q26	The pain limits my ability to do things.	4	3	3	4	4	4	6	1	
Q27	I feel powerless.	4	4	3	4	4	4	6	1	
Q28	The pain exhausts me.	4	4	4	3	4	4	6	1	
Q29	I miss the days when I could make things clear.	3	3	4	4	2	4	5	0.83	

**Note:**

S-CVI = The total I-CVI/Number of items.

### Pilot study results

The findings revealed that while 28 participants found the content of the scale items clear, they encountered difficulties in understanding the method of answering. The original questionnaire employed a dichotomous scoring system, wherein responses were scored as 1 for correct and 0 for incorrect. After extensive deliberation within our research group and obtaining expert approval, it was determined that a modification to Likert’s 4-point rating scale would be implemented, where a score of 3 represented “always,” a score of 2 represented “most times,” a score of 1 represented “rarely,” and a score of 0 represented “never.” Subsequently, a follow-up preliminary survey was conducted, indicating that patients reported clarity regarding the content and method of answering each item on the scale.

### Participant characteristics

A total of 160 questionnaires were distributed, all of which were returned. Of these, 150 were deemed valid, yielding a valid response rate of 93.75%. Consequently, 150 patients diagnosed with systemic sclerosis were included in the study. The mean age of the participants was 53.05 (11.31) years. The sample comprised 35 males (23.3%) and 115 females (76.7%). Regarding education, 67 patients (44.7%) had completed junior high school or lower. Marital status indicated that 127 patients (84.7%) were married. Employment status showed that 28 patients (18.7%) were employed, while 19 (12.7%) resided with their spouses. Additionally, 45 patients (30.0%) were from rural areas. For further demographic details, see Table 3.

**Table 3:** Demographic data of study subjects (*n* = 150). The age of the patients and the duration of illness were presented as mean ± standard deviation, whereas other indicators were expressed as percentages.

Demographics characteristics		Number of participants/Mean ± SD	(%)	Demographics characteristics		Number of participants/Mean ± SD	(%)
Sex	Male	35	23.3	Household incomes *per capita*	<5,000	46	30.7
	Female	115	76.7		5,000–10,000	77	51.3
Age		53.05 ± 11.31			>10,000	27	18.0
Marital status	Not married	23	15.3	The resident manner	Stay alone	0	0
	Married	127	84.7		Stay with parents	18	12.0
Permanent residence	Rural area	45	30.0		Stay with spouse	19	12.7
	City	105	70.0		Stay with parents and spouse	73	48.7
Educational status	Middle school	67	44.7		Stay with offspring	35	23.3
	Senior middle school	30	20.0		Others	5	3.3
	Junior college	5	3.3	Disease-Related characteristics			
	Bachelor’s degree or above	39	26.0	SSc Subtype	Diffuse	89	59.3
	Illiteracy	9	6.0		Limited	61	40.7
Medical insurance	Public expense	5	3.3	Disease duration		6.85 ± 7.22	
Medical insurance	Resident medical insurance	94	62.7	Pulmonary involvement	No involvement	46	30.7
	Rural cooperative medical service	45	30.0		Involvement	104	69.3
	Others	6	4.0				

### Item analysis results

The correlation coefficient (r) values (*P* < 0.001) between the questionnaire’s total score and each item’s scores are referred to in Table 3. The critical ratio (CR) for each item was utilized as the test index to evaluate the discriminative power among the questionnaire items. The results indicated that the differences between the high and low groups on each item were statistically significant (*P* < 0.05), as presented in Table 4.

**Table 4:** The correlation coefficient (r) values and the critical ratio (CR) of SScQoL (*P* < 0.001).

Items	r	CR	95 percent confidence interval	
			Lower limit	Lower limit
Item1	0.646	9.785	2.507	1.659
Item2	0.654	12.252	2.367	1.706
Item3	0.660	12.140	2.654	1.906
Item4	0.757	14.834	2.728	2.082
Item5	0.732	16.915	2.824	2.229
Item6	0.623	11.286	2.450	1.716
Item7	0.470	10.054	2.409	1.613
Item8	0.646	9.409	2.341	1.524
Item9	0.479	6.097	2.139	1.086
Item10	0.531	6.395	2.092	1.099
Item11	0.516	7.747	2.105	1.254
Item12	0.512	8.768	2.378	1.498
Item13	0.477	7.634	2.203	1.291
Item14	0.633	6.987	2.264	1.260
Item15	0.475	6.991	2.206	1.228
Item16	0.709	6.996	2.046	1.140
Item17	0.499	11.726	2.551	1.811
Item18	0.403	15.480	2.769	2.138
Item19	0.449	8.239	2.314	1.414
Item20	0.468	5.299	1.809	0.821
Item21	0.728	5.097	1.845	0.809
Item22	0.538	6.440	2.091	1.103
Item23	0.401	6.733	2.159	1.174
Item24	0.617	13.706	2.561	1.912
Item25	0.338	8.852	2.295	1.452
Item26	0.549	4.629	1.891	0.754
Item27	0.575	11.255	2.512	1.757
Item28	0.725	3.978	1.689	0.562
Item29	0.433	12.861	2.475	1.812

### Reliability testing results

The Cronbach’s α coefficient, test-retest reliability, and split-half reliability of the SScQoL are presented in Table 5.

**Table 5:** The Cronbach’s α coefficient, test-retest reliability, and split-half reliability of the SScQoL.

	Total	Emotion	Function	Social	Pain	Sleep
Cronbach’s α	0.922	0.942	0.890	0.870	0.853	0.756
Test-retest reliability	0.969	0.961	0.956	0.852	0.906	0.710
Split-half reliability	0.876	0.922	0.875	0.878	0.853	0.756

### Content validity results

Based on the assessment by six experts, the I-CVI of the scale ranged from 0.83 to 1.00, and the S-CVI/Ave was 0.97, indicating that each item effectively reflects the quality of life in patients with systemic sclerosis. Refer to Table 2 for expert scoring and calculation of content validity.

### Construct validity results

**Figure 2:**
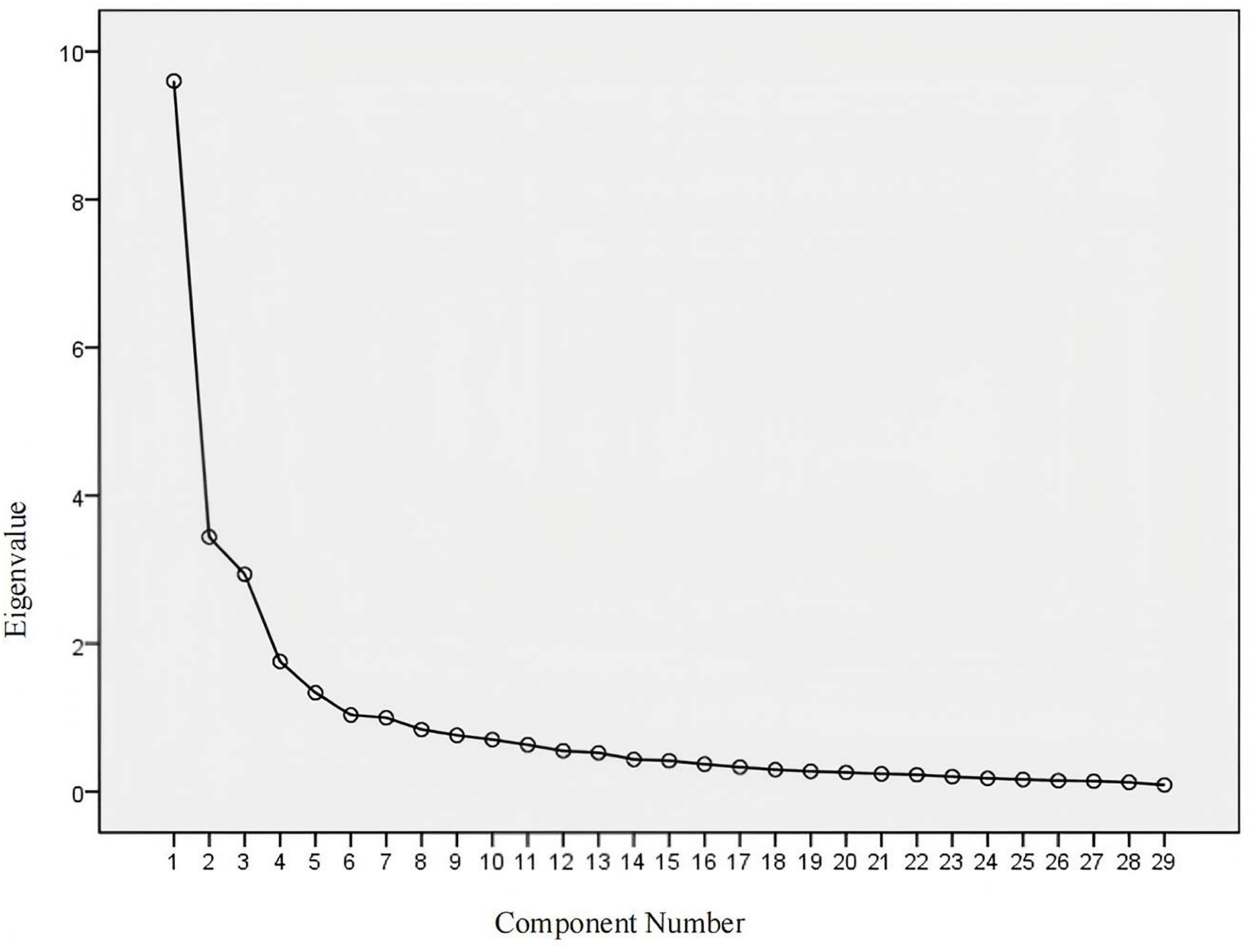
The scree plot. Analysis performed using exploratory factor analysis in SPSS.

Exploratory Factor Analysis. An exploratory factor analysis was performed on the 29 items of the Chinese version of the SScQoL. The KMO value was 0.840, and Bartlett’s sphericity test yielded a chi-square value of 2,928.599 (df = 406, *p* < 0.001). The analysis employed the principal component method and varimax rotation. Common factors with eigenvalues ≥ 1 were extracted, yielding five common factors aligned with the original scale. The cumulative variance explained by these factors was 65.761%, and all item loadings on their respective factors exceeded 0.400. Consequently, all items were retained. The factor loadings and cumulative variance explained for the Chinese version are presented in Table 6. The scree plot is illustrated in Fig. 2.

**Table 6:** The factor loadings and cumulative variance explained of SScQoL.

Items	Emotion	Function	Social	Pain	Sleep
1. I have to think carefully about everything I do.	0.808	0.104	0.053	0.045	0.034
2. I have consistently kept this condition, systemic sclerosis, at the forefront of my mind.	0.802	0.073	0.097	0.037	0.005
4. I find my current situation frustrating	0.800	0.115	0.197	0.006	0.198
5. I feel quite uncomfortable when I cannot accomplish tasks.	0.788	0.103	0.250	0.038	0.000
3. The thought of letting others down is causing me concern.	0.785	0.073	0.102	0.022	0.042
17. The things that never bothered me have started to make me worry.	0.785	0.110	0.031	0.076	0.060
24. I feel remorseful for falling ill.	0.784	0.129	0.084	0.266	0.081
8. I feel like I am constantly facing challenges every moment.	0.747	0.015	0.024	0.057	0.086
29. I miss the days when I could make things clear.	0.728	0.113	0.196	0.280	0.050
7. I cannot expect what my tomorrow will be like.	0.687	0.053	0.140	0.044	0.146
6. I sometimes feel frustrated.	0.674	0.048	0.130	0.168	0.233
27. I feel powerless.	0.652	0.265	0.032	0.254	0.047
18. How life is now is different from what it used to be.	0.620	0.341	0.262	0.029	0.030
12. My hands are not as dexterous as they used to be.	0.124	0.853	0.100	0.013	0.143
19. The situation is overwhelming for me.	0.114	0.818	0.017	0.112	0.093
25. I managed to finish washing up with incredible difficulty.	0.126	0.810	0.122	0.202	0.010
14. I should make it a point to take more frequent breaks and rest.	0.198	0.763	0.034	0.207	0.026
22. Doing household chores can sometimes be challenging.	0.064	0.753	0.079	0.193	0.052
15. Every activity poses its challenges.	0.138	0.714	0.161	0.050	0.154
13. It has limited my interpersonal relationships.	0.133	0.061	0.828	0.066	0.101
16. I avoid attending certain social occasions because I feel awkward.	0.153	0.046	0.811	0.054	0.016
23. I have to give up some of my hobbies.	0.150	0.027	0.780	0.113	0.024
10. It has a significant impact on my social life.	0.125	0.097	0.776	0.062	0.091
21. The current situation makes me feel quite lonely.	0.059	0.121	0.684	0.163	0.058
11. The health of people around me has been impacted.	0.174	0.232	0.637	0.130	0.104
28. The pain exhausts me.	0.095	0.079	0.094	0.861	0.037
26. The pain limits my ability to do things.	0.131	0.074	0.157	0.850	0.127
20. Having a poor sleep has a significant impact on me.	0.158	0.106	0.079	0.164	0.839
9. My situation means that my sleep has been disrupted.	0.171	0.161	0.238	0.000	0.827
Variance contribution rate	33.112	11.858	10.127	6.058	4.605
Cumulative Variance Contribution Rate	33.112	44.971	55.098	61.156	65.761

### Ceiling effect and floor effect results

The results of the ceiling and floor effects are shown in Table 7.

**Table 7:** The results of the ceiling effect and floor effect.

Demention (Item count)	Ceiling effect	Floor effect	Is it acceptable?
Emotion(13)	10%	10%	Yes
Function(6)	17.3%	12.7%	Yes (The ceiling effect is slight)
Social(6)	9.3%	10%	Yes
Pain(2)	29.3%	35.3%	No (The floor effect is significant)
Sleep(2)	30.7%	22%	No (The ceiling effect is significant)
Overall questionnaire(29)	0.7%	0.7%	Yes

## Discussion

The study shows that the quality of life questionnaire for patients with systemic sclerosis (SSc) is a valid and reliable instrument for assessing the quality of life. The localized version of the questionnaire has strong psychometric properties, including high internal consistency, test-retest reliability, and good content and construct validity. These results align with those of the original questionnaire. Additionally, the questionnaire features a manageable number of items, uses clear and comprehensible language, and includes a 4-point Likert scale (0–3), where higher scores indicate worse quality of life. Therefore, the localized SSc-QoL is an effective and specialized tool for evaluating the quality of life in SSc patients.

In the pretest stage, 28 participants indicated difficulties in comprehending the secondary scoring method used in the questionnaire. It may be attributed to the relatively low discriminative power of the secondary scoring method, which hinders its ability to capture subjective feelings or subtle differences among respondents. As a result, some participants may experience confusion while answering or find it challenging to identify an option that precisely aligns with their circumstances. After discussions within the research team and consultations with experts, the scoring method was revised to a Likert 4-point scale. This adjustment aligns with the scoring approach utilized in the German version of the questionnaire (Kocher et al., 2021). The revised German version of the SSc-QoL employs the Likert 4-point scoring method and has been validated for its reliability and validity. The original binary (yes/no) scoring approach was modified to a 4-point Likert scale (0–3 points) to capture subtle variations in patients’ psychological conditions more effectively. This revision improved the scale’s sensitivity and discriminative capacity while mitigating potential response bias associated with forcing participants to choose between only two extreme options. Preliminary validation findings suggest that the revised scale offers a more clinically relevant categorization of symptom severity. The psychometric implications of this scoring approach will be further investigated in future validation studies.

This study investigated the reliability and validity of a quality-of-life questionnaire designed for patients with systemic sclerosis (SSc) by employing Classical Test Theory (CTT), as opposed to the Item Response Theory (IRT) utilized in the original development of the questionnaire. The main reason for choosing classical test theory (CTT) over item response theory (IRT) in this study is the relatively small sample size of patients with systemic sclerosis (SSc), which makes IRT less suitable due to its requirement for larger samples. CTT is also computationally simpler, well-suited for evaluating the questionnaire’s overall reliability and validity, and involves fewer, less strict assumptions, aligning better with the study’s objectives and data conditions. Although these two theoretical frameworks differ, this study’s findings match the original study’s results, showing the questionnaire has good reliability and validity. This consistency indicates that the questionnaire maintains strong psychometric properties across different theories, supporting its use across frameworks and cultural contexts.

The pain dimension shows a ceiling effect, likely due to overly simple item design, which leads most participants to score in the high range and makes it hard to distinguish between moderate and severe pain. Future research will include more detailed pain descriptions to improve scale sensitivity. The sleep dimension shows a floor effect, suggesting current items cannot detect mild sleep issues, with most scores clustering in the low range. It may stem from cultural misunderstandings caused by vague item wording. To enhance measurement accuracy, we plan to refine the item language through qualitative interviews and add specific sleep indicators, such as sleep latency and nighttime awakenings.

This study has two main strengths. First, it includes a relatively large sample size, essential for studying systemic sclerosis, a rare disease. Second, the patients were recruited from medical centers in northern and southern China, enhancing the geographical diversity and representativeness of the findings. The study also has several limitations. First, data collection was limited to two hospitals, which may affect the sample’s representativeness. Second, due to the rarity of the disease, the sample size remained small, preventing confirmatory factor analysis. Third, the study did not compare the questionnaire with other validated tools, such as the SF-36, and lacked external validation. Additionally, the face validity was not formally assessed, which would have provided valuable insights into its clarity and relevance. Our next step is to validate the Systemic Sclerosis Quality of Life Questionnaire through confirmatory factor analysis, criterion-related validity testing, face validity, and external validation using a larger sample.

## Conclusions

In conclusion, since its development, the SSc-QoL scale has been widely used in international research. This study translated the scale into Chinese and confirmed its content validity, clear expression, and strong cultural adaptability for the target population. The scale takes only 5 to 8 min to complete and shows excellent reliability and validity, making it a valuable tool for healthcare professionals to assess the quality of life in patients with systemic sclerosis. However, further research is needed to enhance its clinical applicability. Future studies could examine its use in intervention trials, such as evaluating the impact of medications or rehabilitation therapies on patients’ quality of life, thus supporting more personalized treatment decisions.

## Supplemental Information

10.7717/peerj.20331/supp-1 Supplemental Information 1 General information and questionnaire data of 150 patients with systemic sclerosis.

10.7717/peerj.20331/supp-2 Supplemental Information 2 The Quality of Life Questionnaire for Systemic Sclerosis.

10.7717/peerj.20331/supp-3 Supplemental Information 3 STROBE checklist.

10.7717/peerj.20331/supp-4 Supplemental Information 4 STARD 2015 Checklist. The STARD 2015 Checklist is a 30-item tool designed to improve the transparency, completeness, and reproducibility of diagnostic accuracy studies by ensuring critical methodological and reporting elements are included.

## Additional Information and Declarations

### Data Availability

The following information was supplied regarding data availability:

The raw measurements are available in the Supplemental File.
