# Supplementary material for: Cross-cultural adaptation and psychometric validation of the Chinese quality-of-life questionnaire for patients with systemic sclerosis
Source: PeerJ. 2025 Nov 19;13:e20331. doi: 10.7717/peerj.20331 (PMC12640127; doi:10.7717/peerj.20331)
Supplement: Supplemental Information 2 [file peerj-13-20331-s002.docx]

Dear patient: Hello! Thank you very much for your participation and cooperation in this survey. The purpose of this survey is to understand the quality of life of patients with systemic sclerosis in order to better guide our clinical work in the future. The survey was divided into two parts: the first part was the general information questionnaire, and the second part was the quality of life questionnaire for systemic sclerosis. The survey will be kept confidential. There will be no right or wrong answers. You can fill in the questionnaire based on your actual situation. Please mark "√" on the corresponding options and do not omit any items. Thank you again for your cooperation!

Part one: General Information Questionnaire:

1. Gender: ① Male ② Female
2. Age____ years
3. Marital status: ① unmarried ② married
4. Occupation: ① student ② employed ③ retired ④ farmer ⑤ Other
5. Religion: ① Buddhism ② Christianity ③ Islam ④ Others ⑤ None
6. Educational attainment: ① Junior high school and below ② High school including technical secondary school ③ Junior college ④ Bachelor's degree and above ⑤ Illiterate
7. Medical insurance: ① Out-of-pocket ② Labor expenses ③ Resident medical insurance ④ New Rural Cooperative Medical care ⑤ Commercial insurance ⑥ others
8. Household per capita income: ① more than 5,000 ② 5,000-10,000 ③ more than 10,000
9. Place of residence: ① rural ② urban
10. Ways of living: ① living alone ② living with parents ③ living with spouse ④ Living with spouse and children ⑤ Live with children ⑥ others 11 .Types of systemic sclerosis: ① systemic ② localized

12. Systemic sclerosis duration____ years

13. Lung involvement: ① None ② Yes Part

Part2.Questionnaire on quality of life in Patients with systemic sclerosis

| **Item** | **3** | **2** | **1** | **0** |
| --- | --- | --- | --- | --- |
| 1.I have to think carefully about everything I do | Always | most times | rarely | never |
| 2.I have consistently kept this condition ，systemic sclerosis, at the forefront of my mind. | Always | most times | rarely | rarely |
| 3. The thought of letting others down is causing me concern. | Always | most times | rarely | rarely |
| 4.I find my current situation frustrating | Always | most times | rarely | rarely |
| 5.I feel quite uncomfortable when I cannot accomplish tasks. | Always | most times | rarely | rarely |
| 6.1 sometimes feel frustrated. | Always | most times | rarely | rarely |
| 7.I cannot expect what my tomorrow will be like. | Always | most times | rarely | rarely |
| 8.I feel like I am constantly facing challenges every moment. | Always | most times | rarely | rarely |
| 9.My situation means that my sleep has been disrupted. | Always | most times | rarely | rarely |
| 10.It has a significant impact on my social life. | Always | most times | rarely | rarely |
| 11.The health of people around me has been impacted | Always | most times | rarely | rarely |
| 12.My hands are not as dexterous as they used to be. | Always | most times | rarely | rarely |
| 13.1t has limited my interpersonal relationships. | Always | most times | rarely | rarely |
| 14.I should make it a point to take more frequent breaks and rest. | Always | most times | rarely | rarely |
| 15.Every activity poses its challenges. | Always | most times | rarely | rarely |
| 16.I avoid attending certain social occasions because I feel awkward. | Always | most times | rarely | rarely |
| 17.The things that never bothered me have started to make me worry. | Always | most times | rarely | rarely |
| 18.How life is now is different from what it used to be. | Always | most times | rarely | rarely |
| 19. The situation is overwhelming for me. | Always | most times | rarely | rarely |
| 20. Having a poor sleep has a significant impact me. | Always | most times | rarely | rarely |
| 21.The current situation makes me feel quite lonely. | Always | most times | rarely | rarely |
| 22.Doing household chores can sometimes be challenging. | Always | most times | rarely | rarely |
| 23.I have to give up some of my hobbies. | Always | most times | rarely | rarely |
| 24.I feel remorseful for falling ill. | Always | most times | rarely | rarely |
| 25.I managed to finish washing up with incredible difficulty. | Always | most times | rarely | rarely |
| 26.The pain limits my ability to do things. | Always | most times | rarely | rarely |
| 27.1 feel powerless. | Always | most times | rarely | rarely |
| 28.The pain exhausts me. | Always | most times | rarely | rarely |
| 29.1 miss the days when I could make things clear. | Always | most times | rarely | rarely |
